# In Silico Analysis of the Subtype Selective Blockage of KCNA Ion Channels through the µ-Conotoxins PIIIA, SIIIA, and GIIIA

**DOI:** 10.3390/md17030180

**Published:** 2019-03-19

**Authors:** Desirée Kaufmann, Alesia A. Tietze, Daniel Tietze

**Affiliations:** 1 Technische Universität Darmstadt, Eduard-Zintl-Institute for Inorganic and Physical Chemistry, Alarich-Weiss Str. 8, 64287 Darmstadt, Germany; kaufmann@chemie.tu-darmstadt.de; 2 University of Gothenburg, Department of Chemistry and Molecular Biology, Wallenberg Centre for Molecular and Translational Medicine, Kemigården 4, 41296 Göteborg, Sweden; alesia.a.tietze@gu.se

**Keywords:** potassium channel, µ-conotoxins, µ-PIIIA, µ-SIIIA, µ-GIIIA, ion channel, toxin interactions

## Abstract

Understanding subtype specific ion channel pore blockage by natural peptide-based toxins is crucial for developing such compounds into promising drug candidates. Herein, docking and molecular dynamics simulations were employed in order to understand the dynamics and binding states of the µ-conotoxins, PIIIA, SIIIA, and GIIIA, at the voltage-gated potassium channels of the KV1 family, and they were correlated with their experimental activities recently reported by Leipold et al. Their different activities can only adequately be understood when dynamic information about the toxin-channel systems is available. For all of the channel-bound toxins investigated herein, a certain conformational flexibility was observed during the molecular dynamic simulations, which corresponds to their bioactivity. Our data suggest a similar binding mode of µ-PIIIA at KV1.6 and KV1.1, in which a plethora of hydrogen bonds are formed by the Arg and Lys residues within the α-helical core region of µ-PIIIA, with the central pore residues of the channel. Furthermore, the contribution of the K+ channel’s outer and inner pore loops with respect to the toxin binding. and how the subtype specificity is induced, were proposed.

## 1. Introduction

Voltage-gated ion channels, such as potassium (K_V_), calcium (Ca_V_), or sodium (Na_V_), mediate the ion flow through the membrane that is essential for various physiological functions. Whereas K_V_ channels are of the utmost importance for the electrical excitability of muscle cells and neurons, sodium selective Na_V_ channels are crucial for the initiation and propagation of action potentials [1].

Unlike Na_V_ channels, which are heterotetramers, K_V_ channels are homotetrameric complexes. Both Na_V_ and K_V_ ion channels consist of six transmembrane helices (S1–S6). The loop-like linker segment that connects the transmembrane helices S5 and S6 is located within the center of the channel’s pore duct, and harbors the selectivity filter [2,3]. Furthermore, the voltage-sensing S4 segments are responsible for the opening of the channel [3,4], which is further controlled by the outer located helical segments S1–S3 [5,6]. As the mechanism of ion-conduction through voltage-gated potassium channels appears to be well understood [4,7], the mechanism of sodium ion conduction through Na_V_ channels is still under intense investigation [1,8,9,10,11,12,13].

The agonistic and antagonistic deactivation of the ion channels form the basis of the analgesic effects, either triggering a constant opening or direct occlusion of the channel [14,15,16]. For example, peripheral Na_V_1.7 channels are essential for mediating the pain signal [17], and are suggested as being a valuable target.

Conotoxins—small, cysteine-rich polypeptides obtained from the venom of marine cone snails—are such ion channel antagonists. Some of these toxins are known to block voltage-gated ion channels by occluding their pores [18,19,20], thus interrupting the signal transmission between neurons. Conotoxins usually consist of 10 to 30 amino acids and are grouped into superfamilies according to their disulfide pattern. µ-Conotoxins, which are known to specifically inhibit Na_V_ channels, are typically 16 to 25 amino acids long and harbor three disulfide bridges, with the so-called “native fold” connecting Cys1–Cys4, Cys2–Cys5, and Cys3–Cys6 (numbered in the order of occurrence in the amino acid sequence) [21,22]. As some of these µ-conotoxins (µ-SIIIA and µ-PIIIA) show some inhibitory activity towards Na_V_1.7, are considered as having a potential for analgesics, as long as they are specific [23,24].

**Figure 1:**
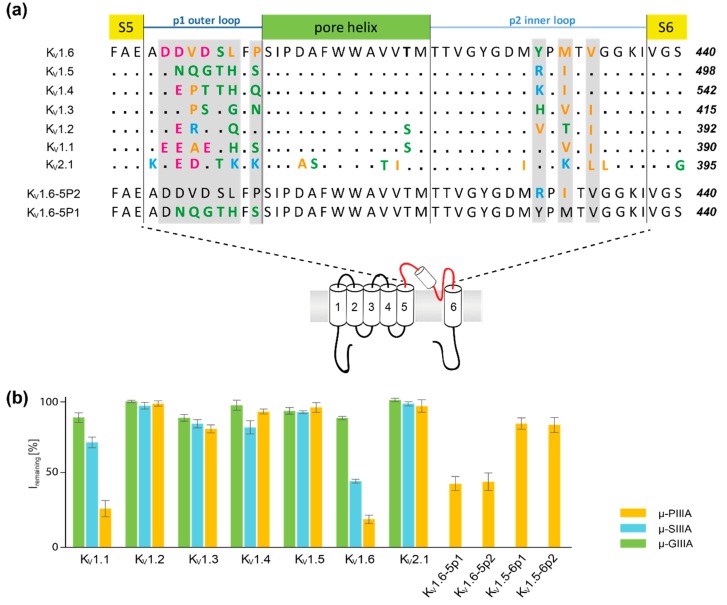
(**a**) Aligned amino acid sequence of the central, toxin-interacting channel region shown for one subunit of the Kv1 family (Kv1.1–Kv1.6), Kv2.1, and of the chimeras Kv1.6-5P2 and Kv1.6-5P1, which were all tested against μ-PIIIA, μ-SIIIA, and/or μ-GIIIA, respectively. by Leipold et al. [1]. Amino acids are coloured according to their physicochemical properties (basic—light blue; acidic—magenta; polar/neutral—green; non-polar polar/hydrophobic—orange). The secondary structure elements are indicated above the alignment. (**b**) Activity rates [%] of μ-PIIIA, μ-SIIIA, and μ-GIIIA on potassium channels Kv1.1–Kv1.6, Kv2.1, and Kv1.5–Kv1.6 chimera channels, as published by Leipold et al. [1]. Lower percentage values of I_remaining_ correspond to higher blocking activities.

Unlike the conotoxin, μ-GIIIA, which exclusively blocks skeletal muscle voltage-gated sodium channel NaV1.4, the conopeptides μ-PIIIA and μ-SIIIA additionally inhibit the neuronal sodium channel NaV1.2. Recently, the latter two were shown to not be exclusively specific for NaV channels [1,25,26,27]. μ-PIIIA and μ-SIIIA were inactive on subtypes KV1.2 to KV1.5, and KV2.1 μ-SIIIA only partially inhibited KV1.1 and KV1.6, while μ-PIIIA blocked both of the channels, unveiling a nanomolar affinity towards them (Figure 1b) [1]. Additionally, Leipold et al. constructed and evaluated the chimeras between KV1.5 and KV1.6, unveiling that the channel block by µ-PIIIA involves the pore regions, whereas the subtype specificity is determined in part by the sequence close to the selectivity filter (P2 inner loop, Figure 1a), but predominantly by the so-called turret domain (P1 outer loop, Figure 1a) [1].

Unfortunately, this clearly limits their analgesic potential. Despite Leipold et al.’s disappointing observations, the high number of well resolved potassium channel crystal structures would certainly allow for a profound and detailed in-silico analysis of µ-conotoxin binding to K_V_ channels. Moreover, the experimental data obtained by Leipold and coworkers might allow us to shed light on the questions of how toxins achieve their subtype specificity on a much higher qualitative level, as this would currently be possible for µ-conotoxin binding at Na_V_.

Thus, this work makes use of powerful state-of-the-art in-silico approaches, investigating the subtype-specific inhibition of potassium channels by μ-conotoxins. Based on the experimental data published by Leipold et al. [1], we performed docking and subsequent molecular dynamics (MD) simulation experiments illustrating the dynamic interplay between μ-conotoxins μ-PIIIA, μ-SIIIA, and μ-GIIIA at the potassium channels K_V_1.1, K_V_1.5, and K_V_1.6, and at two chimeric channel constructs. In this context and for reasons of comparison, µ-SIIIA was investigated as a partially channel blocking semi-active system, while µ-GIIIA was investigated as an inactive candidate. In particular, the different pore blocking modes—full or partial pore coverage—were of special interest, and were intended to achieve more insight into the origin of the remaining currents, especially observable for semi-active systems. Our studies revealed that the centric µ-PIIIA residues were responsible for the blockage events on K_V_1.1 and K_V_1.6, which is compliant with studies of µ-PIIIA on Na_V_ channels. More specifically, our results indicate that residues Lys9, Arg12, Arg14, and Gln15, located within the toxin center of μ-PIIIA, are responsible for blocking the K_V_1.1 and K_V_1.6 channels. So far, these residues have also been reported to be essential for the blockage of sodium channels in different binding modes [19,28,29]. We further observed that the remaining currents may arise from an insufficient pore coverage or from (coincident) the increased dynamics of the toxin or specific toxin residues.

## 2. Methods

### 2.1. Homology Modelling

The YASARA molecular modeling program (Yasara structure, Vers. 18.3.23, Yasara Biosciences GmbH, Vienna, Austria) [30,31] was used to create a homology model of the Kv1.6 potassium channel. The modeling parameters used for the complete process were as follows.

On the basis of the complete Kv1.6 amino acid sequence, 89 possible templates were identified, running three subsequent PSI-BLAST (NCBI—National Center for Biotechnology Information, U.S. National Library of Medicine, Bethesda, MD, USA) iterations [32]. A position specific scoring matrix (PSSM) from UniRef90 [33] was extracted, for which the Protein Data Bank (PDB) was then searched for a match (hits with an E-value below the cutoff of 0.5) in a second step. Altogether, four hits (PDB-IDs 2R9R-B, 3LNM-B, 3LUT-B, and 2A79-B) were identified from the 89 structures available as suitable modeling templates for Kv1.6. To aid in alignment correction and loop modeling, a secondary structure prediction for the target sequence had to be obtained. This was achieved by, again, running PSI-BLAST in order to create a target sequence profile, and then feeding it to the PSI-Pred secondary structure prediction algorithm [34]. To help align the target and template sequences, a target sequence profile was created from a multiple sequence alignment, which in turn was built from the related sequences from the PSI-BLAST obtained the UniRef90 sequences in the first step.

For the four template PDBs, altogether, 29 models were generated based on the alternative alignments of the target and the respective template protein sequence. Side chains were added using YASARA’s implementation of SCWRL3 [35], and were fine-tuned by considering the electrostatic, knowledge-based packing iterations and solvation effects. The hydrogen bond network was optimized [36], and each model was then subjected to an unrestrained energy minimization with explicit water molecules by simulated annealing employing the YASARA2 force field [31].

The 29 models were ranked by their overall quality Z-scores. In addition, YASARA created a hybrid model by combining the individual models’ best parts. As this hybrid model was ranked the best, it was used as the final channel model. A more detailed description of YASARA’s homology modeling protocol can be found online (http://yasara.org/homologymodeling.htm).

Employing the Kv1.6 homology model, further model structures were generated for Kv1.1 and Kv1.5, and for the chimera channels “Kv1.6-5P1” and “Kv1.6-5P2”, respectively. For the Kv1.6-5P1 homology model residues of the “p1”-loop of Kv1.6 were substitute by the residues of the “p1”-loop of Kv1.5 (Figure 1) maintaining the backbone secondary structure.For the Kv1.6-5P2 homology model the residues of the “p2” -loops of the Kv1.6 channel were substituted by the residues of the “p2”-loops of Kv1.5 (Figure 1) while maintaining the backbone secondary structure. In order to yield the Kv1.5 and Kv1.1 homology model the sequence of the p1 and p2 loop of the original Kv1.6 homology model (Figure 1) was changed accordingly, maintaining the secondary structure of the parent structure. Finally, all additional models were energy-minimized for further use.

The voltage-sensor domains of the modeled Kv1 channels were omitted from any further steps, as they are not involved in the µ-conotoxin binding studied in this work.

### 2.2. Docking

The toxin channel binding was predicted by docking the NMR structures of μ-PIIIA [37] (PDB ID: 1R9I), μ-SIIIA (BMRB- Biological Magnetic Resonance Bank ID: 20023), and μ-GIIIA (PDB ID: 1TGC) on the potassium channel Kv1.1, Kv1.5, and Kv1.6, and the chimera homology models, using the Easy Interface of the HADDOCK online platform [38,39,40] (https://haddock.science.uu.nl/services/HADDOCK2.2/haddockserver-easy.html), a web service known to be suitable for handling more complex peptide ligand structures [39].

For the docking process, residue regions, which are part of the channel’s upper surface, as well as all of the toxin residues, were defined as “active”, as they were assumed to be able to form contacts with the toxin (Figure 1). For “passive” channel residues, we defined all of the residues that were either on the “active” ones’ surface, or that surrounded them within a radius of a maximum 6.5 Å within the system.

From the docking results, the best scoring structure from the highest scoring complex cluster was selected for further analysis (Table 1).

Additionally, each structure was rescored using AutoDock Vina (Oleg Trott, Molecular Graphics Lab, La Jolla, CA, USA) with default parameters (Table 1) [41]. The setup was done with YASARA [30].

### 2.3. Molecular Dynamics Simulations and Energy Minimizations

The MD simulations were performed using the YASARA molecular modeling software (Yasara structure, Vers. 18.3.23, Yasara Biosciences GmbH, Vienna, Austria) [42].

As the HADDOCK web interface cannot handle γ-pyroglutamic acid, glutamic acid was used for the docking routine, and prior to the MD simulations, was re-converted to γ-pyroglutamic acid, followed by a subsequent energy minimization step.

The simulations were performed within a cuboid simulation cell employing YASARA’s implemented simulation routine for the simulation of membrane proteins in a lipid membrane environment. Phosphatidyl-ethanolamine (PEA) was used to mimic the native lipid membrane environment during our simulations.

The energy minimizations and refinement simulations of the toxin channel complex were performed as an unrestrained all-atom molecular dynamics simulation for 0.5 to 1 µs in explicit water (TIP3P) using the PME method [43], in order to describe long-range electrostatics at a cut-off distance of 8 Å in physiological conditions (0.9% NaCl, pH 7.4 [44]), at a constant temperature (298 K) using a Berendsen thermostat, and with constant pressure (1 bar). The charged amino acids were assigned according to the predicted pKa of the amino acid side chains from the Ewald summation, and were neutralized by adding counter ions (NaCl) [44]. In order to increase the simulation performance, a multiple time step algorithm, together with a simulation time step interval of 5 fs [42], was chosen using the AMBER14 force field [45,46] and by removing the high frequency bond and angle vibrations of the hydrogen atoms, employing constraints through the LINCS [47] and SETTLE [48] approach. The simulation snapshots were saved every 250,000 fs. The YASARA2 [31] force field was used for energy minimization by simulated annealing, including the optimization of the hydrogen bond network [36] and the equilibration of the water shell, until system convergence was achieved.

The molecular graphics were created using YASARA (Yasara structure, Vers. 18.3.23, Yasara Biosciences GmbH, Vienna, Austria, www.yasara.org) and POVRay (Persistence of Vision Raytracer Pty. Ltd., www.povray.org).

## 3. Results and Discussion

According to the experimental results obtained by Leipold et al., a detailed in-silico analysis of the binding mode and the dynamics of μ-PIIIA, μ-SIIIA, and μ-GIIIA on the K_V_1-channel members K_V_1.1, K_V_1.5, and K_V_1.6, and on the two K_V_1.6/1.5 chimeras was performed.

Firstly, HADDOCK dockings were performed employing the NMR structure of the respective toxin and a homology model of the channel target (for more details on the homology models see method section). The best scoring HADDOCK result was used for further analysis, and was additionally rescored using Vina AutoDock, revealing toxin binding energies with a remarkable correlation with respect to the toxin’s activity rates (Table 1) [1]. In order to attain more accurate descriptions of the toxin binding and toxin dynamics when bound to its target, all of the docked structures were equilibrated through molecular dynamics simulations in a membrane environment. This might be of special importance, as the docked toxin structures were all centred in the middle of the pore (Figure 1), which was hard to interpret in terms of their bioactivity, as they did not only show a full pore block (named active thereafter), but also suggested partly blocked ion-channel pores (named semi-active thereafter) [1].

At this point, it shall be noted, that in case of the non-pore blocking (named inactive thereafter), the toxin-channel systems docking results and simulation data might be somewhat awkward and not straight forward to interpret. Nevertheless, for reasons of comparison and completeness, these data were also shown and analysed.

### 3.1. Toxin Dynamics and Cluster Analysis

In order to equilibrate the docked toxin-channel structures, we performed a molecular dynamics simulation in a membrane environment until a nearly linear behaviour of the toxins root mean square deviation (RMSD) (Cα-atoms) was observed, resulting in simulation times varying between 0.5 and 1.0 µs. During these simulations, we noticed a significant dynamic of the toxin on the channel surface for some toxin-channel systems (see Supplementary Figure S1), according to the toxin’s RMSD.

These observations suggested a unique, stable mode of channel blockage, while pointing out a more diverse behavior for semi- and in-active systems. More precisely, we identified two different binding modes for the semi-active systems, which are represented by the more stable and less fluctuating systems of μ-PIIIA-K_V_1.6-5P1 and μ-SIIIA-K_V_1.6, and by the more dynamic and less stable μ-PIIIA-K_V_1.6-5P2 (Supplementary Figure S1). Likewise, two different modes of toxin movement were identified for the two inactive toxin-channel systems. For K_V_1.5-bound µ-PIIIA, the toxin displacement occurred more gradually, whereas for μ-GIIIA, it was moving more rapidly on the channel surface (Supplementary Figure S1).

Closer inspections of the toxin movements with respect to the four channel subunits elucidated the stated differences within the semi- and in-active systems, clearly unveiling a toxin movement towards a channel subunit for μ-PIIIA on K_V_1.6-5P2 (semi-active) and μ-GIIIA on K_V_1.6 (inactive) (Supplementary Figures S2 and S3). More specifically, μ-PIIIA moved towards the channel’s p1 outer loop of subunit II (SII), and simultaneously away from the outer p1 loop of the opposite channel subunit I (SI) (Supplementary Figures S2 and S3), which is most likely triggered by the Y429R mutation in the p2 loop near the selectivity filter, simultaneously reducing the pore blockage. In contrast, the semi-active systems μ-PIIIA-K_V_1.6-5P1 and μ-SIIIA-K_V_1.6, as well as μ-PIIIA-K_V_1.5 (inactive), did not show a toxin movement towards any subunit, maintaining a more center-positioned state on the pore (Supplementary Figures S2 and S3).

Assuming that the extent of the pore blockage is given by the different adopted toxin orientations on the pore, we aimed at completing further investigations on the corresponding interactions arising at such an equilibrium. It is obvious that such key interactions, depending on their stability, can hold the toxin in a specific place on the channel, and can allow for the maintenance of a stable conformation. The resulting individual toxin positioning on the pore and its overall stability will finally constitute a stronger or weaker channel blockage.

For the detection of system-wise representative snapshots, we developed and applied a selection protocol for the parsing of a periodically pre-filtered subset of 11 simulation snapshots (in the interval from 0 ns up until 500 ns, spaced at 50 ns), based on a combination of two cluster search methods (for more details, see Supplementary Materials).

**Figure 2:**
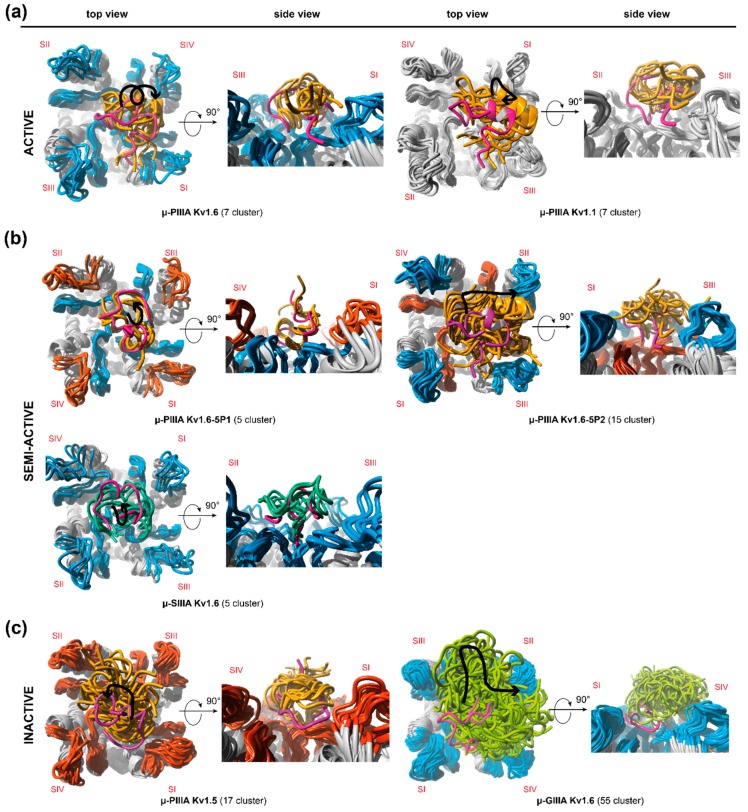
Secondary structure representation of the identified clusters, which were derived from the molecular dynamics simulations showing one representative per main cluster (4 Å toxin RMSD threshold) for (**a**) active, (**b**) semi-active, and (**c**) inactive systems. The black traces with arrowhead in the top view figures indicate the toxin movement on the channel surface throughout the simulation. The total numbers of identified clusters are given for each toxin-channel system in brackets. Colouring: μ-PIIIA—orange; μ-SIIIA—turquois; μ-GIIIA—green. Channel loops: Kv1.5—red; Kv1.6—blue; Kv1.1—grey. Channel subunits are indicated by Roman numerals (SI–SIV).

So far, combined cluster analyses together with a final revising step have provided a refined and computationally underpinned method for the systematised selection of representative simulation states. Thus, arbitrary token decisions considering representative snapshot selections out of large volume of data can be verified or even rejected. Figure 2 shows the individually chosen snapshots as energy-minimized structures, and gives a preliminary indication of the correlation between the channel blockage, structural features, and positioning of the toxin on the channel.

Apart from the RMSD analyses, it was obvious that the toxin-channel systems, where the toxin moves away from a central starting position, intrinsically hold higher flexibilities as extracted from the simulations per atom b-factors (Supplementary Figure S6).

Further examinations of the different simulation cluster representatives (Figure 2) equally confirm this inference, pointing out a higher number of cluster representatives for the more flexible systems and a lower number of cluster representatives for toxin-channel systems, which reside in the centre of the pore (Figure 2, Supplementary Figure S6). Furthermore, in total, the inactive systems were found to have more cluster representatives, that is, a higher fluctuation as active and semi-active systems (Figure 2). In addition, our afore-stated suggestion of a unique binding mode (and related flexibility) for active systems was endorsed by the equal amount of resulting cluster representatives for μ-PIIIA on K_V_1.1 and K_V_1.6 (Figure 2, Supplementary Figure S6). The systems’ different flexibilities were equally reflected by our own clustering approaches, which were further used for the detection of representative snapshots (Supplementary Table S2).

Contrarily, the semi-active toxin-channel systems (μ-SIIIA-K_V_1.6 and μ-PIIIA-K_V_1.6-5P1) exhibited a similar low overall fluctuation, suggesting similar binding stabilities compared to the active ones (μ-PIIIA-K_V_1.6/K_V_1.1), and accordingly, showed a similar number of cluster representatives over the whole simulation (Figure 2, Supplementary Figure S6). Nevertheless, μ-PIIIA-K_V_1.6/K_V_1.1 had a better pore coverage with equally distributed contacts to all four of the channel subunits, whereas μ-SIIIA-K_V_1.6 and μ-PIIIA-K_V_1.6-5P1 clearly had a preference to one side of the channel (Supplementary Figure S6). In the case of μ-PIIIA-K_V_1.6-5P1, this tendency must have been triggered by the mutations in the outer loop. At the same time, when the P2 loop of K_V_1.6 was mutated towards the toxin insensitive K_V_1.5 channel, an even more sideward orientation of the toxin was observed, which was presumably induced by the newly inserted positively charged Arg429. Altogether, the experimental activities could be rationalized from the simulation data, even though one would expect somehow higher flexibilities or dynamics for μ-SIIIA-K_V_1.6 and μ-PIIIA-K_V_1.6-5P1 in order to understand the remaining currents for these semi-active systems. Most likely, the rather short simulation times (up to 1 µs) were insufficient to fully uncover all of the aspects of the pore blocking. This was especially true for the binding of μ-PIIIA at the toxin insensitive channel K_V_1.5. Here, one would expect no binding, or at least a relatively fast toxin unbinding, as observed for μ-GIIIA on K_V_1.6. Despite the fact that unbinding was not observed during our 1 µs simulation, μ-PIIIA was only bound to the center of the pore, and only showed very little contact with the pore surface, being clearly different from the situation of toxin binding at the active and semi-active systems, supposing that μ-PIIIA was about to dissociate from the pore soon.

### 3.2. Analysis of Channel-Toxin Interactions

In the following, we will give a concise description of the toxin–ion channel key interactions based on a final representative, which was selected from the cluster analysis.

**Figure 3:**
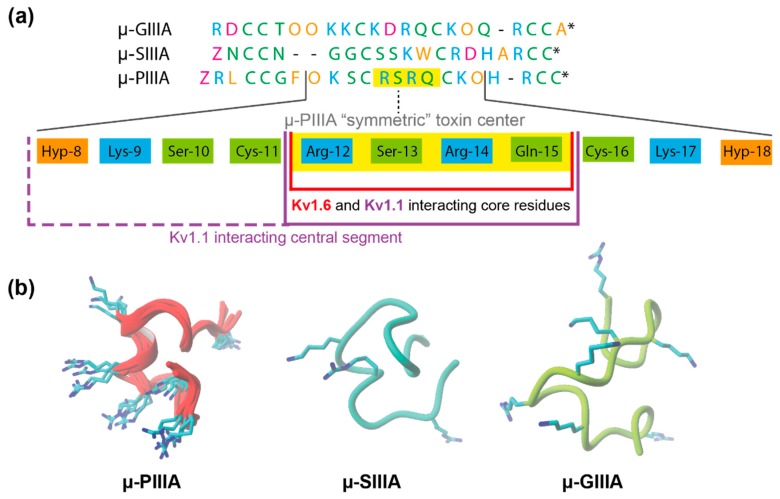
(**a**) Alignment of the µ-conotoxins µ-GIIIA, µ-SIIIA, and µ-PIIIA. Residues are coloured by their physicochemical properties (purple—acidic; cyan—basic; orange—nonpolar/hydrophobic; green—polar/neutral). The “symmetric” µ-PIIIA centre is given below the alignment, highlighting the central interacting motif of µ-PIIIA in yellow. (**b**) Structures of µ-PIIIA (superposition of all of the docked structures), µ-SIIIA, and µ-GIIIA when bound at the K_V_ channel after HADDOCK docking.

**Figure 4:**
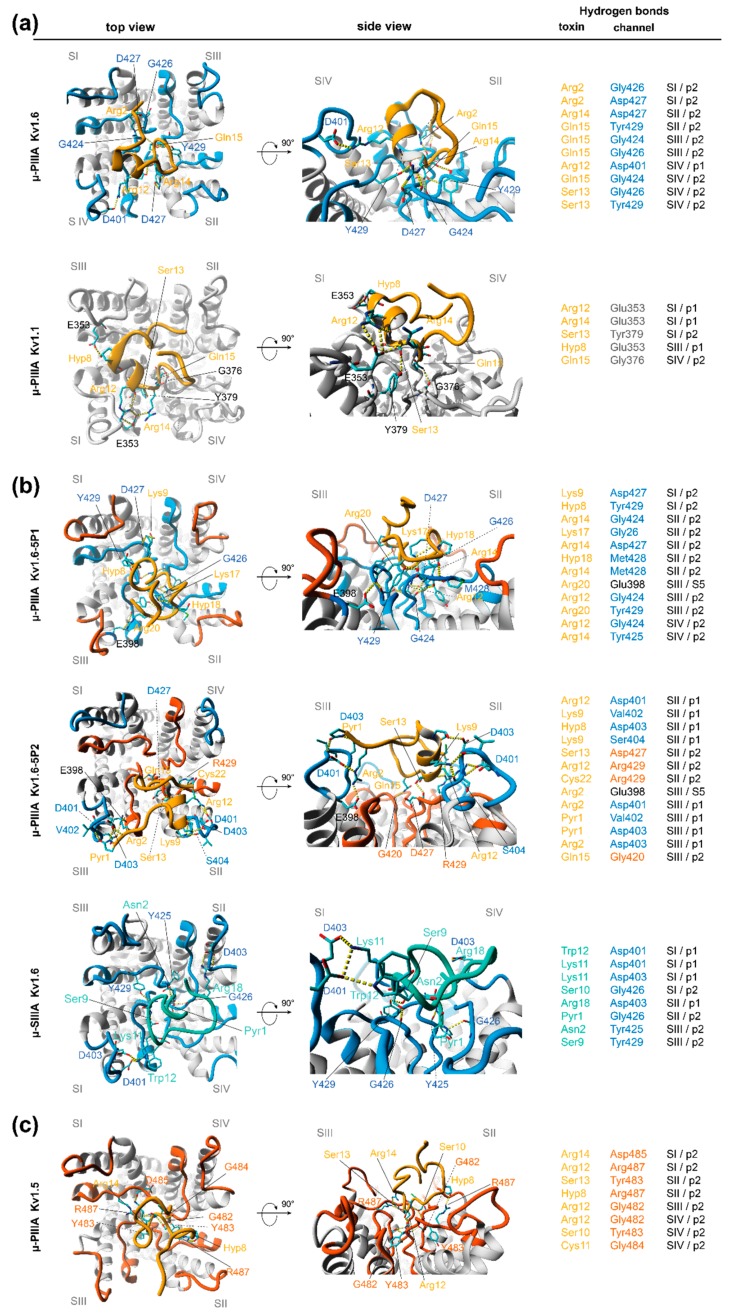
Representative binding poses of µ-PIIIA and µ-SIIIA at different K_V_ channels indicating hydrogen bond interactions (yellow dotted lines) for (**a**) active, (**b**) semi-active, and (**c**) inactive systems. H-bond interactions are listed the correspondingly. Because of its high dynamics, the individual interactions of µ-GIIIA on Kv1.6 are not shown. Colouring: μ-PIIIA—orange; μ-SIIIA—turquois. Channel loops: Kv1.5—red; Kv1.6—blue; Kv1.1—no grey. Channel subunits are designated by Roman numerals (SI–SIV).

μ-PIIIA-Kv1.1/1.6: As already mentioned earlier in the text, a very similar µ-PIIA orientation on K_V_1.6 and on K_V_1.1 was revealed. Primarily, this effective and stable pore block appeared to arise from interactions of the centrally located toxin residues (Figure 3a), which were evenly distributed towards at least three of the four subunits (SII–SIV). For μ-PIIIA bound to K_V_1.6, the pore block was even further stabilized by the interactions of Arg-2 with the inner P2 loop residues of subunit I (SI) (Figure 4). Interestingly, for K_V_1.6-bound µ-PIIIA, the hydrogen bonds were primarily formed towards the residues of the inner pore loops (P2), whereas for μ-PIIIA bound to K_V_1.1, more residues of the outer loops (P1) were addressed (Figure 4).

According to our simulation data, a major role of the blockage of K_V_1.1 and K_V_1.6 seems to be attributed to Arg12 and Arg14, forming hydrogen bonds towards the glutamic (Glu353/SI in Kv1.1) or aspartic acid (Asp401/SIV in K_V_1.6) of the outer P1 loops of the channels, or with Asp427/SII (K_V_1.6) of the inner loop of K_V_1.6 (Figure 4), which led us to conclude that the switch from Asp in K_V_1.6 to Glu in K_V_1.1 did not affect the structure and physicochemical composition of the outer P1 loop of the channel, resulting in similar toxin binding modes for µ-PIIIA. The further centrally located neutral serine Ser13 was interacting with the equally neutral glycine or larger tyrosine residues near the selectivity filter (Gly426/SIV and Tyr429/SIV of K_V_1.6 and Tyr379/SI of K_V_1.1, Figure 4). In addition, Ser10 was further able to form an additional hydrogen bond towards His355/SIII of the outer P1 loop in K_V_1.1, which was Leu in K_V_1.6. Another key role of the specific blockage of K_V_1.6 can be attributed to Gln15 forming interactions primarily with the outer glycines of the selectivity filter and Tyr429/SII, which were located on the pore surface (Figure 4). This interaction was also present in K_V_1.1-bound µ-PIIIA (Figure 4).

In summary, the µ-PIIIA key hydrogen bond interactions with K_V_1.6 were primarily formed towards the residues near the selectivity filter (Gly and Tyr) of the inner P2 loops, and Asp residues in the outer P1 loops. In case of Kv1.1, the P1 loops contained Glu353 instead of Asp401, which led to an increased number of contacts of µPIIIA with the P1 loops. The µ-PIIIA H-bond donor residues were comprised of Arg12, Ser13, Arg14, and Gln15 for binding at K_V_1.6 and K_V_1.1, and additionally, by Lys9 and Ser10 for K_V_1.1 (Figure 4).

Interestingly, these key residues were all localized within the α-helical centre of μ-PIIIA, containing basic and neutral amino acids in an alternating symmetric pattern (Figure 3a). According to the physicochemical properties of the amino acids of the µ-PIIIA sequence, this symmetric toxin centre consists of 11 amino acids, reaching from Hyp-8 to Hyp-18, comprising a K--R-R--K-like motif. In this context, it was noticed that µ-SIIIA lacks such a motif or a similar symmetric motif in the corresponding region (Figure 3a), whereas µGIIIA unveiled a remotely similar motif (KK-K-R—K). Apparently, the α-helical conformation of this region in µ-PIIIA ensures that the positively charged residues are pointing towards the channel surface, rather than being buried or hidden by the fold of the toxin, while the corresponding regions of µ-SIIIA and µ-GIIIA lacked such a helical structure (Figure 3b), assuming that this feature further strengthens the stability of the interacting residues in the bound state.

**Figure 5:**
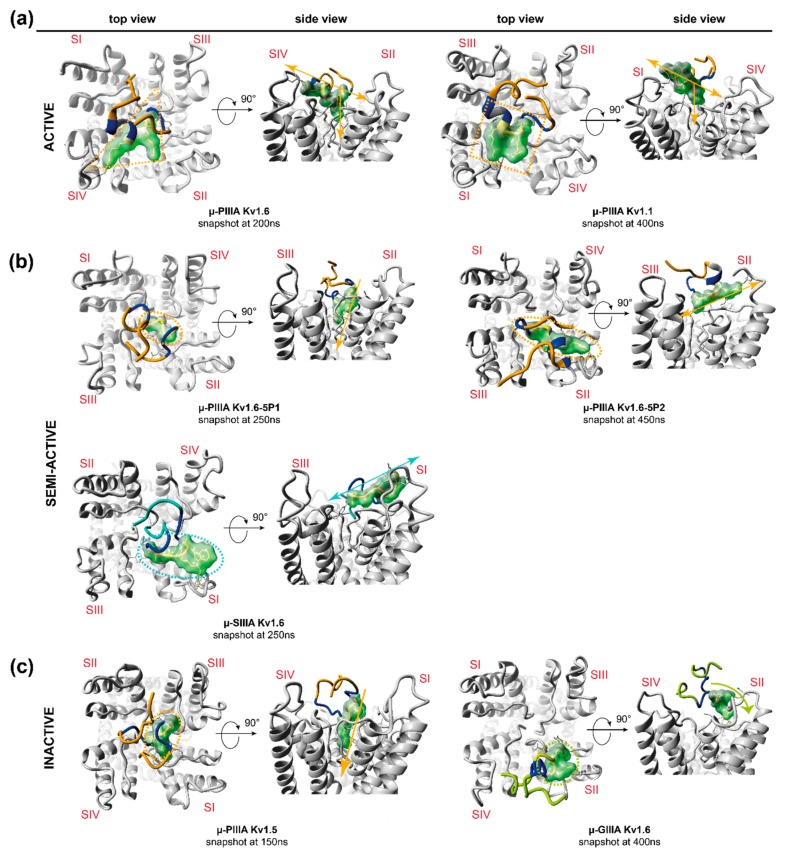
Overview of the channel bound toxin state regarding the orientation of the symmetric toxin centre for (**a**) active, (**b**) semi-active, and (**c**) inactive systems. Structures of the representative snapshots are shown in secondary structure representation (channels are coloured grey, residues which account for the µ-PIIIA core motif Arg-12–Gln-15 are shown as yellow sticks, together with their molecular surface (transparent light green). Dashed lines indicate the overall channel coverage by the (binding) motif-corresponding core. The motif’s surrounding area, corresponding to the symmetric µ-PIIIA central segment Hyp-8–Hyp-18, is coloured dark blue, and the remaining toxin endings are coloured individually to the systems (µ-PIIIA on Kv1.6—red; µ-PIIIA on Kv1.1—violet; µ-PIIIA on Kv1.6-5P1—dark orange; µ-PIIIA on Kv1.6-5P2—light green; µ-SIIIA on Kv1.6—sun-yellow; µ-PIIIA on Kv1.5—green; µ-GIIIA on Kv1.6—cyan). Delineated arrows, equally coloured to the toxin endings, indicate the overall orientation of the core-motif regions with respect to the channel surface. Channel subunits are indicated by Roman numerals (SI—SIV).

We also noticed that the symmetric µ-PIIIA centre was almost parallel to the channel surface when bound to K_V_1.1 and K_V_1.6 (Figure 5). In contrast, a “kinked” conformation of the overall central segment in the semi- and in-active systems (including Kv1.6-bound µ-SIIIA) was observed, suggesting a reduced pore coverage (Figure 4 and Figure 5). Again, this supports the uniqueness of µ-PIIIA’s blocking mode towards K_V_1.1 and K_V_1.6. Lastly, the stability of the µ-PIIIA-K_V_1.1 and -K_V_1.6 interactions was also mirrored by the overall per residue RMSFs of the concerning µ-PIIIA residues displaying the lowest value for the symmetric toxin centre (Supplementary Figure S5).

Interestingly, similar observations regarding the significance of the symmetric toxin centre for channel blockage by μ-PIIIA were also reported for µ-PIIIA binding to Na_V_1.4, suggesting Arg14, Arg12, Lys17, and Arg20 as the key interacting residues [18,28]. Another study revealed multiple binding modes for µ-PIIIA binding to Na_V_1.4, suggesting that Lys9 or Arg14 protrud into the channel pore [29], of which the later binding mode was strikingly similar to the K_V_1.6-bound µ-PIIIA (see Supplementary Figure S7a,b), thus strongly supporting the functional importance of the central toxin segment.

µ-PIIIA K_V_1.6-5P1: Even though the selected snapshot showed a blocked pore when µ-PIIIA was bound to K_V_1.6-5P1, it was completely lacking the necessary and stabilizing interactions of the µ-PIIIA residues with the P1 loops, as they were exchanged with P1 loops of K_V_1.5 (Figure 5). Additionally, the toxin orientation was found to be similar to the low affinity pose when bound to the toxin-insensitive K_V_1.5 channel. Thus, the outer loop modification into a mainly polar/neutral environment triggered a toxin displacement, and a coincident increase of interactions to the central pore, resulting in a different toxin binding pose compared with K_V_1.1 and K_V_1.6 (Figure 4 and Figure 5). Furthermore, the symmetric toxin centre was tilted by ~ 90° with respect to the channel plane (Figure 5), now facing towards a single channel subunit and lacking any H-bond interactions to Q15 and S13 (Figure 4 and Figure 5). However, sole contacts to the inner P2 loops and the lack of H-bonds to the P1 loops might lead to a reduction in the binding affinity, thus resulting in an incomplete pore block as observed by Leipold and co-workers [1].

µ-PIIIA Kv1.6-5P2: In contrast, when the polar/neutral Tyr429 in K_V_1.6 was mutated into a positively charged Arg in Kv1.6-5P2, the crucial interactions of µ-PIIIA residues S13 and Q15 with the centre of the pore, as observed for µ-PIIIA binding at K_V_1.6 and K_V_1.1, were eliminated (Figure 4). As already hypothesized above, the positive charge of R429 enforced a toxin binding pose, which was again different from what was observed before. Consequently, S13 and Q15 moved towards the unaltered P1 loops, forming H-bonds with the Asp (D427/SII) residues in this region (Figure 4 and Figure 5). Furthermore, the tendency of µ-PIIIA to contact the more acidic residues of the P1 outer loops was reflected by the interactions of R12, K9, and O8 with D401/SII, D403/SII, and S404/SII, thus resulting in a partial pore covering. This interpretation was further supported by the high degree of conformational flexibility on the pore surface (Figure 2, Supplementary Figure S6).

µ-PIIIA Kv1.5: With respect to the identified interactions of µ-PIIIA with K_V_1.6 and how they were altered when K_V_1.6 was stepwise modified towards the toxin-insensitive K_V_1.5 channel, µ-PIIIA was found in a different orientation, and lacking most of these interactions when bound to K_V_1.5. The resulting upright binding position of the core segment (Arg12–Ser13–Arg14) of µ-PIIIA was similar to the toxin orientation at K_V_1.6-5P1 (Figure 4 and Figure 5). The plethora of H-bonds of the symmetric toxin centre were formed only with the residues next to the selectivity filter motif (Figure 4), covering a much smaller portion of the channel surface compared with the other toxin-channel systems, suggesting a low-affinity binding pose. This interpretation is further supported by the lowest Vina AutoDock-derived binding energy (Table 1) of all of the toxin-channel systems analysed in this work, and by the high degree of local per residue flexibility outside the µ-PIIIA’s core region (Supplementary Figure S6).

Altogether, it seems that the µ-PIIIA binding at the K_V_-channels is mainly defined by the residues of the inner P2 loop, which strongly influences the overall orientation of the toxin on the channel surface, enabling the necessary stabilizing interactions with the outer P1 pore loops.

µ-SIIIA Kv1.6: As µ-SIIIA is similarly active (semi-active) to µ-PIIIA on the Kv1.6-1.5 chimeras, we also analysed the µ-SIIIA binding at K_V_1.6 (Figure 1). In contrast to µ-PIIIA, µ-SIIIA had a somewhat different sequence, which lacked a symmetric toxin centre of µ-PIIIA (Figure 3). Interestingly, our analysis unveiled that the central region of µ-SIIIA (Ser9 to Trp12), which corresponds to the symmetric centre of µ-PIIIA, was mainly forming H-bonds with the central pore region, as it was observed for µ-PIIIA binding at K_V_1.65P2 (Figure 5). Similar to the Gln15 of µ-PIIIA, Ser9 and Ser10 now addressed Tyr429/SIII and Gly426/SI of the inner P2 loops (Figure 4). Furthermore, the H-bonds between Lys11, Trp12, and Arg18 with Asp401/SI and Asp403/SI of the outer P1 loops, were proposed to stabilize a more sideward oriented µ-SIIIA binding pose, whereas Gln1 and Asn2 were forming H-bonds with Gly426/SII and Tyr425/SIII, with the centre of the pore rationalizing an incomplete channel block (Figure 4 and Figure 5). Interestingly, Asp401 was equally addressed by the centrally located Arg12 of µ-PIIIA, further supporting a similar binding pose of K_V_1.6-bound µ-SIIIA with K_V_1.6-5P2-bound µ-PIIIA (Figure 4 and Figure 5). The experimentally observed incomplete block of K_V_1.6 by µ-SIIIA was further rationalized by the conformational and local flexibilities of the outer P1 loops, leading to a reduced stability of the binding pose (Supplementary Figure S6), as observed for other cases discussed in this work [1].

µ-GIIIA Kv1.6: Lastly, unlike µ-PIIIA at K_V_1.5, our equilibration simulation revealed a very high motion and dynamic of µ-GIIIA on the channel surface, which clearly reflects its low binding affinity and inability to bind to K_V_1.6, as revealed by Leipold et al. [1].

## 4. Conclusions

Our in-silico data provided insight into the dynamics and binding states for the binding of the µ-conotoxins PIIIA, SIIIA, and GIIIA at the voltage-gated potassium channels of the K_V_1 family. So far, their different activities can only adequately be understood when dynamic information about the toxin-channel systems is available. For all of the channel bound toxins investigated herein, a certain conformational flexibility was observed during the molecular dynamics simulation (Figure 2), which most likely accounts for the remaining currents of these systems. Some of the semi-active and inactive toxin-channel systems (µ-PIIIA K_V_1.6-5P2, and µ-GIIIA K_V_1.6) showed significantly higher conformational flexibilities rationalizing their incomplete pore block, together with a clearly visible incomplete pore coverage or sideward orientation of the toxin. In contrast, the less flexible semi-active toxin-channel system (µ-PIIIA K_V_1.6-5P1) cannot be fully rationalized by this criterion, but clearly lacks the interactions identified at the more active toxin channel systems.

So far, our data suggest a unique and similar binding mode of µ-PIIIA at K_V_1.6 and K_V_1.1, in which the plethora of hydrogen bonds are formed by the α-helical core region of µ-PIIIA with the central pore residues of the channel. Furthermore, the binding mode of µ-PIIIA at Na_V_1.4 was found to be similar to the µ-PIIIA orientation, when bound to K_V_1.6 and K_V_1.1, supporting the importance of the centric µ-PIIIA residues (Supplementary Figure S7a,b) [28,29]. Also, Arg12 and Arg14, which were shown to stabilize the pore-blocking position of µ-PIIIA on K_V_1.6 and on K_V_1.1, were also considered to be important for µ-PIIIA binding at Na_V_1.4, as revealed from earlier MD studies [29]. Moreover, the herein predicted orientation of µ-PIIIA at K_V_1.6 was found to be similar compared to the experimentally determined orientation of the structurally related µ-conotoxin KIIIA when bound to Na_V_1.2 (pdb 6J8E), suggesting a unique pore blocking motif for µ-conotoxins (Supplementary Figure S7c) [11].

Interestingly, the insertion of the pore loop residues of the µ-PIIIA-insensitive K_V_1.5 channel into K_V_1.6 resulted in a reorientation of the toxin and a clearly sideward oriented µ-PIIIA core region. A similar sideward orientation was also observed for the corresponding core region of µ-SIIIA in the channel bound state. Altogether, our data suggested that an effective pore block can only be achieved if the toxin is capable of addressing the inner and the outer pore loops, whereas for µ-PIIIA binding at K_V_1.6, K_V_1.5 and the respective chimeras of the composition of the inner pore loop (P2) mainly determine the orientation of the toxin, which is then further stabilized by the outer pore loops (P1). This stabilizing interaction is mainly mediated through the hydrogen bonds of the toxin’s positively charged Arg and Lys residues with the negatively charged Asp (K_V_1.6) or Glu (K_V_1.1) of the outer pore loops, and, here, predominantly with the third Asp (D403) (K_V_1.6) or the corresponding Glu353 (K_V_1.1), which are located closer to the centre of the pore than the other D/E residues of the loop.

Concerning the subtype specificity of µ-PIIIA among the K_V_1 family members, toxin binding does not tolerate positively charged amino acids in either of the pore loops (Figure 1), as is the case for K_V_1.6, K_V_1.3, and K_V_1.1. Most likely, this enforces a strong reorientation and repulsion of the toxin, as concluded from our data for µPIIIA binding at K_V_1.6-5P2. As K_V_1.3 lacks the important third Asp residue in the outer P2 pore loop, µ-PIIIA can only properly bind to K_V_1.6 and K_V_1.1.

However, the D- and E-rich outer P1 pore loops of K_V_1.6 and K_V_1.1 are rather small, requiring a compactly folded toxin (like µ-PIIIA), for which R/K residues can orient towards the channel surface and the outer P2 loops simultaneously, thus partly explaining the inactivity of µ-GIIIA (Figure 3).

Lastly, our in-silico toxin binding study strongly supports the importance of the dynamic information about the toxin-channel systems and the conformational space the toxin can sample in its channel-bound state, even though our molecular dynamic-based equilibration step is not suitable to unveil the full conformational landscape of toxin binding.

Furthermore, as most of the toxin binding poses in our study differ from their initial docked state, we strongly recommend a combined approach of docking and molecular dynamics simulation concerning the in-silico analyses of protein–ligand systems and their coincident evaluation.

Structural data can be provided upon reasonable request.

## Figures and Tables

**Table 1:** Summary of the scoring values for the best scoring µ-conotoxin-channel systems obtained from docking and re-scoring.

		HADDOCK Z-Score	HADDOCK Score	Vina Score (kcal/mol)
μ-PIIIA Kv1.6	active	−1.0	174.3 ± 8.7	10.5
μ-PIIIA Kv1.1	active	−1.4	202.1 ± 5.9	9.5
μ-PIIIA Kv1.6-5P1	semi-active	−1.4	178.2 ± 14.0	9.7
μ-PIIIA Kv1.6-5P2	semi-active	−0.9	196.0 ± 12.7	10.0
μ-SIIIA Kv1.6	semi-active	−1.3	231.1 ± 14.7	10.2
μ-PIIIA Kv1.5	inactive	−1.6	202.6 ± 10.5	8.5
μ-GIIIA Kv1.6	inactive	−1.7	187.1 ± 14.0	8.0

## Supplementary Materials

The following are available online at https://www.mdpi.com/1660-3397/17/3/180/s1, Figure S1: Toxin RMSD, Figure S2: Toxin distances to the outer loops p1, Figure S3: Toxin distances to the inner loops p2, Figure S4: Schematic representation of clustering method, Figure S5: Per residue RMSF summary, Figure S6: B-factor coloring of the interacting toxin and channel residues, Figure S7: Comparison if µ-PIIIA binding Table S1: RMSD to the corresponding docked structure used as start for MD calculations, Table S2: Main stages of the representative snapshot selection process.
