# Supplementary material for: In Silico Analysis of the Subtype Selective Blockage of KCNA Ion Channels through the µ-Conotoxins PIIIA, SIIIA, and GIIIA
Source: Mar Drugs. 2019 Mar 19;17(3):180. doi: 10.3390/md17030180 (PMC6471588; doi:10.3390/md17030180)
Supplement: Supplementary file 1 [file marinedrugs-17-00180-s001.pdf]

Supporting Information

In silico analysis of the subtype selective blockage of KCNA ion channels through the µ-conotoxins PIIIA, SIIIA and GIIIA

Desirée Kaufmann,[1] Alesia A. Tietze[2] and Daniel Tietze[1]*

[1]Technische Universität Darmstadt, Eduard-Zintl-Institute for Inorganic and Physical Chemistry, Alarich-Weiss Str. 8, 64287 Darmstadt, Germany, kaufmann@chemie.tu-darmstadt.de
[2]University of Gothenburg, Department of Chemistry and Molecular Biology, Wallenberg Centre for Molecular and Translational Medicine, Kemigården 4, 412 96 Göteborg, Sweden, alesia.a.tietze@gu.se
* Correspondence: tietze@chemie.tu-darmstadt.de

1. Molecular dynamics simulation

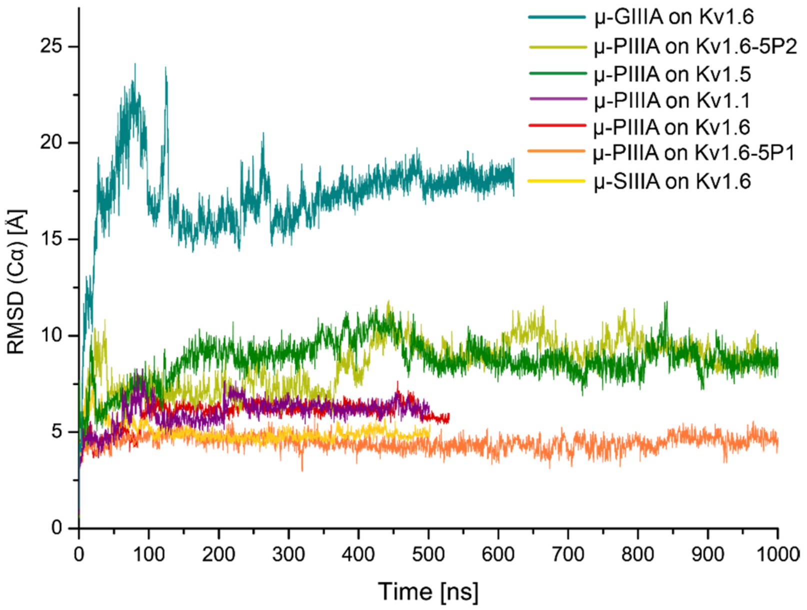

Figure S1: toxin RMSD over the whole simulation; toxin RMSD traces up to 1 µs for semi-active systems µ-PIIIA on Kv1.6-5P1, µ-PIIIA on Kv1.6-5P2 and for the inactive system µ-PIIIA on Kv1.5.

Table S1: RMSD to the corresponding docked structure used as start for MD calculations

	selected snapshot at time [ns]	RMSD from start [Å]
**µ-PIIIA on Kv1.6 at 200ns**	*200*	6.066
**µ-PIIIA on Kv1.1 at 400ns**	*400*	6.077
**µ-PIIIA on Kv1.6-5P1 at 250ns**	*250*	4.462
**µ-SIIIA on Kv1.6 at 250ns**	*250*	4.652
**µ-PIIIA on Kv1.6-5P2 at 450ns**	*450*	10.064
**µ-PIIIA on Kv1.5 at 150ns**	*150*	8.914
**µ-GIIIA Kv on 1.6 at 400ns**	*400*	17.471

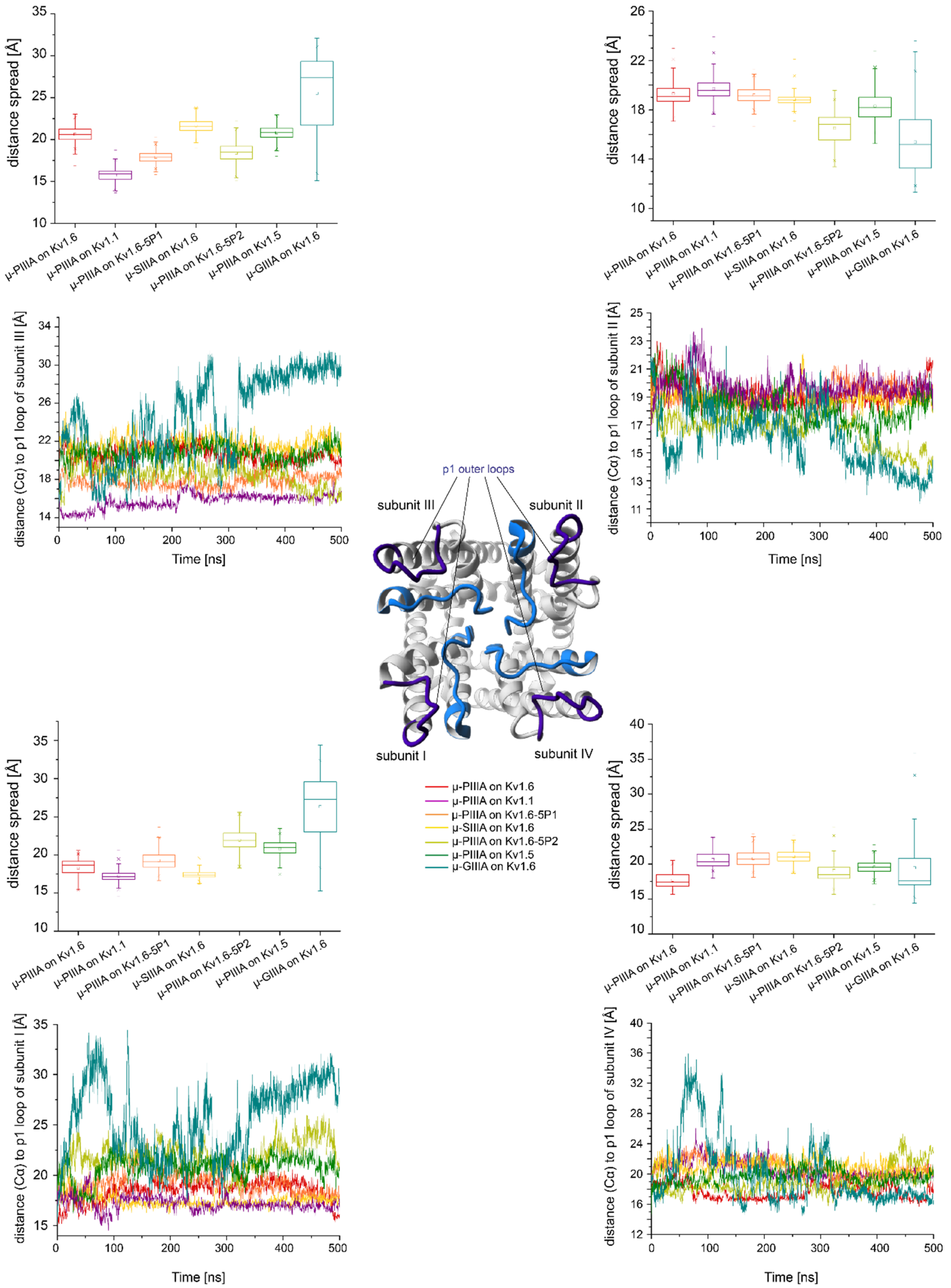

Figure S2: RMSD traces showing the movement, i.e. changing distance of the toxin mass centre with respect to the outer loops (p1) of the individual channel subunits (p1 outer loops – dark blue, p2 inner loops – light blue).

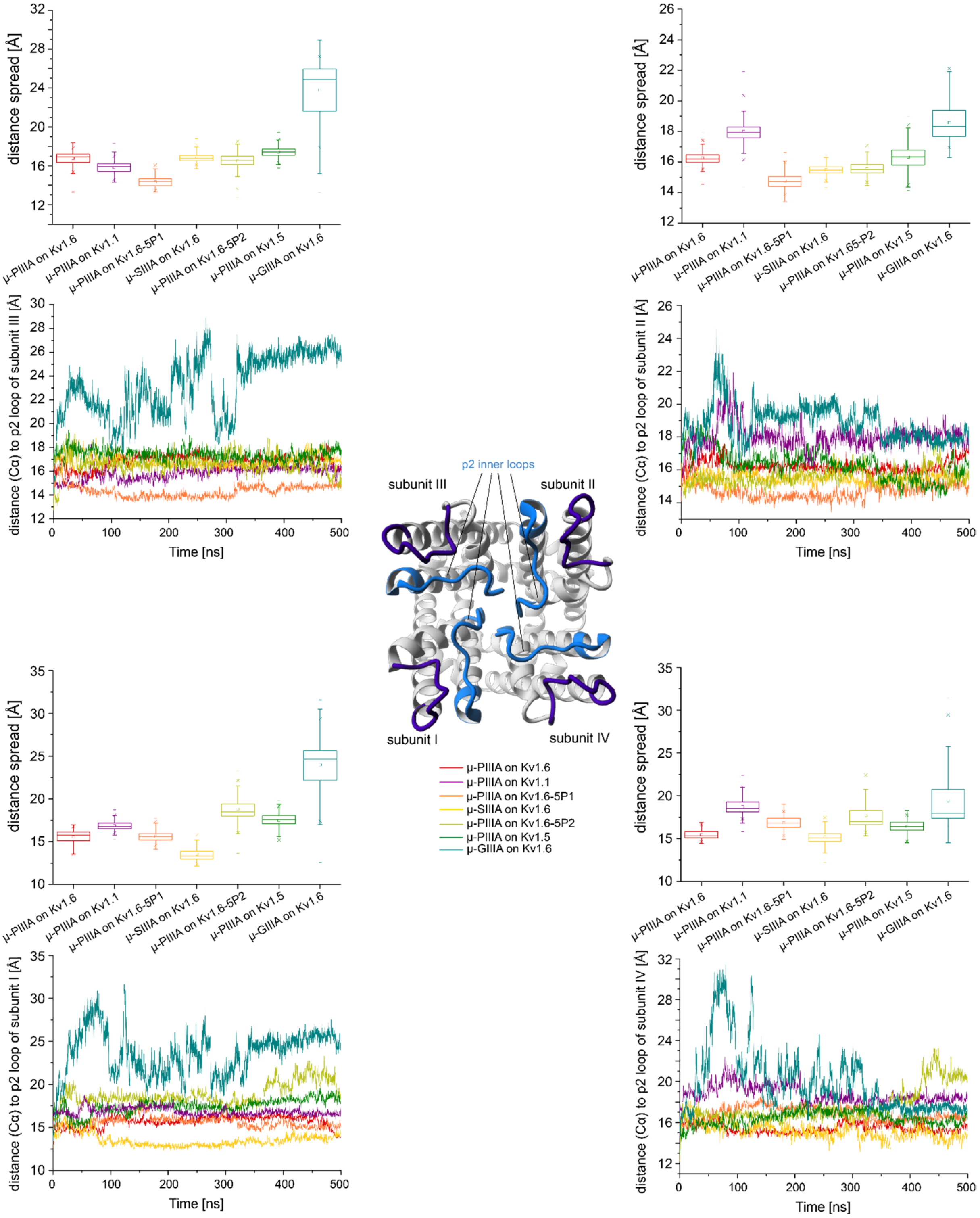

Figure S3: RMSD traces showing the movement, i.e. changing distance of the toxin mass centre with respect to the inner loops (p2) of the individual channel subunits (p1 outer loops – dark blue, p2 inner loops – light blue).

2. Combined clustering for the representative toxin-channel structure selection

i) Method

For the selection of representative snapshots of each simulation we applied a combination of two clustering approaches described below. Prior to the clustering applications, all data sets were subdivided into eleven snapshots respectively, each with an equal spacing of 50ns, as the starting point for the clustering. Hence, the first snapshot is at the start simulation time of 0ns and the eleventh corresponds to simulation snapshot at 500ns.

Cluster Analysis 1:

At first, we extracted a set of snapshots in which the toxin entities deviate by less than 3 Å RMSD from the average toxin structure. This set is defined as the main cluster according to cluster analysis 1. This "clustering" approach naturally only filters a single cluster as it searches for a data subset satisfying one criterion. The remaining data is not further considered, so the stability and dynamics of a system are directly represented by its determined main cluster's size. The corresponding results are depicted in ***Table S2*** (2nd column).

In the following we will use the term "MCL1" to designate the main cluster 1, obtained by approach 1.

Cluster Analysis 2:

To refine the representative selection process, we implemented and applied a graph-traversing approach as illustrated in ***Figure S4***. The algorithm is explained based on a graph representation of the eleven preselected snapshots. This would in theory result in a calculation of all pair-wise distances between each pair of nodes, i.e. pair of toxin simulation snapshots, accompanied by a polynomial computing effort of O(n^2) in a worst case scenario, due to the number of edges = $\binom{n}{k} = \frac{n(n-1)}{2}$ in a complete graph (yielding 55 node distances in our case of eleven snapshots).
However, we try to circumvent this calculation effort by chronologically considering the nodes and calculating a node's distances to all remaining nodes – corresponding to a decision tree. A node will be counted as a cluster member (of the currently considered node instance), if its RMSD to the currently considered node is < 3 Å. The threshold was set to maintain consistencies with the threshold set for clustering method 1.
The next not yet assigned node will depict the considered node defining a newly instantiated cluster for the further iteration. Already assigned and considered nodes are of course not considered in any further iteration in order to avoid redundant calculations of pair-wise distances. This results in a reduced number of pair-wise distance calculations – more specifically in n-1 (according to O(n)) distance calculations corresponding to a linear effort in a best case scenario. A transitive relation of a close distance of a pair of nodes, both fulfilling the criterion to be assigned to a common cluster, is provided.
It needs to be mentioned that in our case, dealing with molecular dynamics simulation data, a chronological way of data analyses or clustering makes sense due to the intrinsic overall data continuity. Results of the second clustering analysis are shown in ***Table S2*** (3rd column).
Despite the outstanding issues and the algorithm's complexity, the second cluster analysis involves several benefits in our opinion, as in contrast to cluster method 1 it also provides and takes into account information about the internal data spread, seen by the number of clusters generated (***Table S2***, 3rd column). This also provides information about the location of the data focus and the intensity of the overall data scattering, not necessarily given by method 1.
As each toxin snapshot is considered and compared to all other ones – not only to the toxin average – the main cluster is detected rather with respect to "real" data and not based on a theoretically calculated size. This approach offers considerable advantages, for example for the precise analysis of very unequally distributed data sets.
The cluster of largest size or the union of the clusters of largest sizes (in case if more than one clusters of same maximum size occur) is finally defined as the main cluster out of the generated clusters (***Table S2***, 3rd column, yellow highlighted set). In the worst case this would result in a non-existent main cluster (empty set) for an extremely fluctuating system in which only size 1 clusters are detected. Stability and dynamics of a system are hence represented by the determined main cluster's size as well as by the concomitant number of clusters generated (and obviously by their respective sizes). In the following we will use the term "MCL2" to designate the main cluster 2, obtained by approach 2.

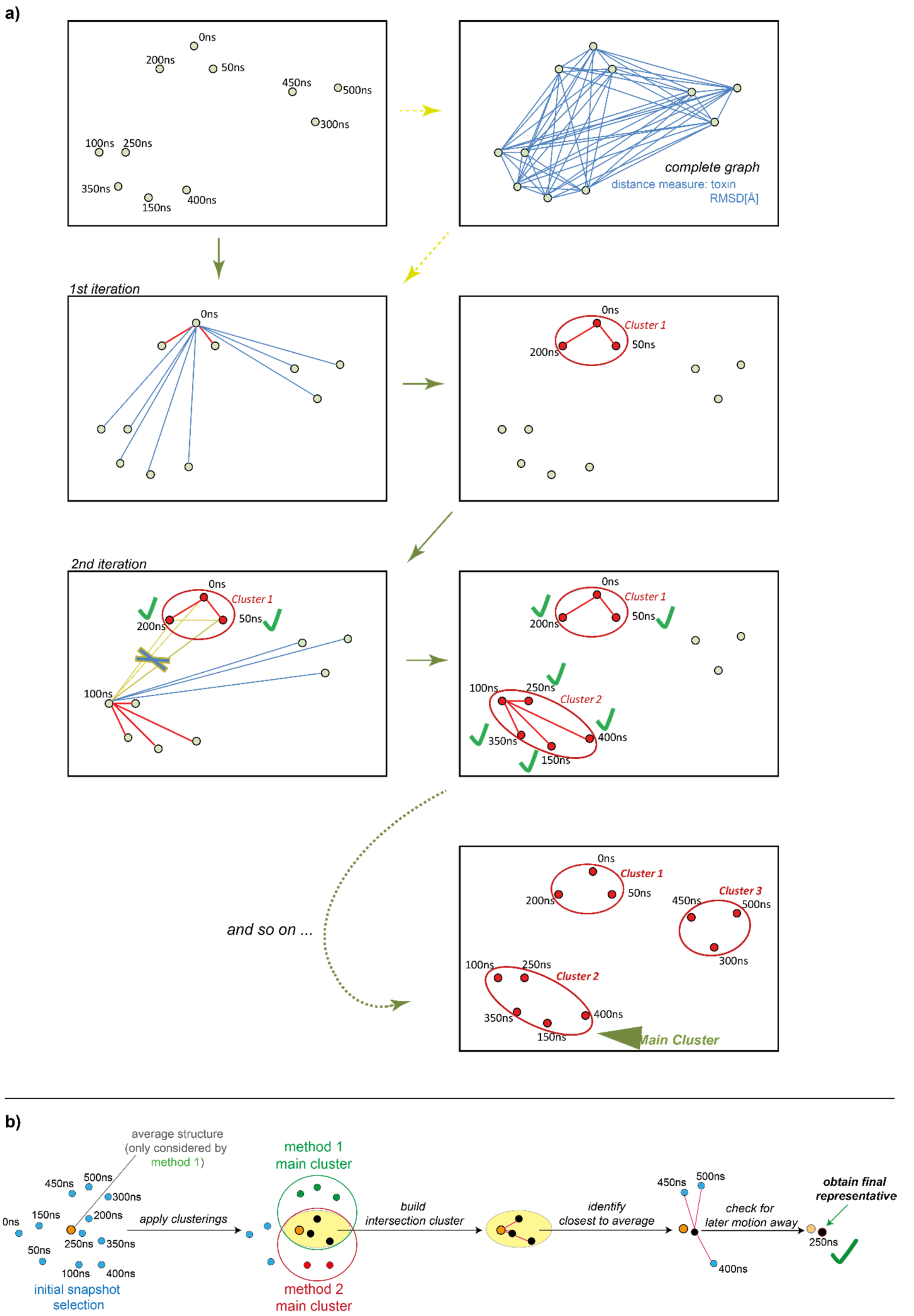

Figure S4: a) schematic representation of clustering method 2 as graph-based view: the clustering is applied on eleven snapshots of a 500ns simulation, equally spaced at 50ns; each snapshot defines a vertex node while the edge lengths are given by the pair-wise toxin RMSD values [Å] between the individual snapshots; the clustering is finished, when each node is assigned to a cluster; the main cluster is determined by the highest number of associated members (vertices) or by the union of the clusters of largest sizes (in case if same maximum size clusters occur). **b)** workflow scheme of the complete selection process combining cluster analyses 1 and 2.

Representative selection

a) *Generating the preselection set*

From both clustering approaches a preselection was generated by (preferably) building the intersection cluster of both methods' resulting main clusters. This was done to ensure a position of high proximity to the mean and preferably concomitant within a region of high data density of the representative that is to be chosen out of this preselection set.

For cases where one of the main clusters results in an empty set and thus the intersection operation would likewise do, such as in the case of µ-GIIIA bound to Kv1.6, we took the union cluster as preselection set for further investigations in order to take the main cluster of at least one of the two methods into consideration. In a (generally possible) worst-case scenario of two empty main clusters and a concomitant empty union set for the preselection, the structure of the general lowest RMSD to the average structure of the eleven initially filtered snapshots would be taken as preliminary representative snapshot for further analyses.

In the following we will use the term "PreSel" to designate the preselection set.

A formal description of building the preselection set is given as follows:

$$PreSel = \begin{cases} MCL\ 1 \cap MCL2, if\ MCL1\ \neq\ \emptyset\ and\ MCL\ 2\ \neq\ \emptyset \\ MCL\ 1 \cup MCL\ 2, \quad else \end{cases}$$

The resulting preselection sets are listed in ***Table S2*** (4th column).

b) *Determine the preliminary representative*

From the (non-empty) preselection set the snapshot of *minimum* RMSD distance to the average structure was chosen as preliminary representative (***Table S2***, 5th column).
Note that this does not necessarily have to be the snapshot from the pre-filtered set of eleven snapshots (or also from the whole simulation) with the generally smallest distance to the average structure, since at least method 2 can determine a main cluster that does not contain this snapshot (for example, in the case of very one-sided, not evenly distributed data). Consequently, it also does not necessarily have to be included in the preselection set.

c) *Revision and final selection*

As among the systems µ-GIIIA Kv1.6 and µ-PIIIA Kv1.6-5P2 we observed the principal detachment towards a channel subunit side at different simulations times (for the inactive µ-GIIIA on Kv1.6 already within the first simulation quarter and for the semi-active µ-PIIIA on Kv1.6-5P2 within around the last simulation quarter, ***Figures S1-S3***), we aimed to trap as representative a snapshot of such an already detached toxin state. As this state is however adopted at later simulation times for semi-active "µ-PIIIA Kv1.6-5P2"-like cases, we needed to perform a final revising step on our preliminary representative in order to check if this snapshot would represent, if necessary, such further detachment, possibly occurring during the later simulation process. For this purpose, we have checked our preliminary representatives for their respective toxin RMSD distances to the toxin structures of the last three snapshots of their corresponding simulation (at simulation times 400ns, 450ns and 500ns).
The threshold to maintain the preliminary representative as final representative snapshot was set to 6.0Å. In case a higher RMSD to at least one of the last three snapshots occurred, the preliminary representative was substituted by that snapshot out of the last three, exhibiting the still lowest RMSD to the preliminary representative. (If several identical minimal values >6.0Å have occurred, one of these concerned snapshots would have been randomly selected as representative).
For µ-PIIIA Kv1.6-5P2 our latest checks resulted in a further revised final representative, replacing the first proposed representative snapshot at 200ns by the snapshot at 450ns.
This replacement also made sense with regard to the corresponding data, since the fluctuation of µ-PIIIA on Kv1.6-5P2 is already expressed in the result of clustering method 2 by the high number of generated clusters – equally to that for inactive systems. (***Table S2***, 3rd column). The corresponding results can be seen in ***Table S2*** (last column).

ii) Results and interpretation

We count on the two presented clustering approaches as both of them are based on the assumption that a stable, equilibrated state can be represented as a main cluster of maximum size out of the 11 pre-selected snapshots and a representative snapshot candidate is consequently most probably present within this stable main cluster. However, as method 1 does not necessarily take into consideration the overall data dispersion nor the location of the main data focus, we create the intersection cluster of them ensuring to consider both aspects, the overall data dispersion as well as the distances of the individual cluster members to the average simulation structure (the latter is even considered until the proposal step for the preliminary representative). In this respect, clustering method 2 can be considered as reconciliation or even completing support of clustering method 1 and we recommend a combination of both approaches in terms of a refined selection process of representatives.

Most of the systems show a more stable constellation around their average structure which is closely located to the median at 250ns. This is shown by the suggestion of a preliminary representative (nearest to the average among the intersection set) that is close to the median (corresponding to snapshot 6 at 250ns), which can be observed for systems covering all levels of activity – namely for µ-PIIIA on Kv1.6, µ-PIIIA on Kv1.6-5P1, µ-SIIIA on Kv1.6, µ-PIIIA on Kv1.6-5P2 and for µ-PIIIA on Kv1.5 (***Table S2***, 5th column). This may be due to the tendency of data collected from nature to assume a more normal distribution with averages close to the centre of the data series ***Table S2*** (2nd – 4th column).

At this point the remarkable identity of the main clusters found by methods 1 and 2 resulting for our µ-PIIIA on Kv.1.6 simulation needs to be pointed out (***Table S2***, 1st row, 2nd – 4th column). It even can be considered as a number-based confirmation of the system's high specificity and stability. Likewise and equally interesting, complete inactive µ-GIIIA on Kv.1.6 shows the precise opposite in this respects, not pointing out any intersection between the results of method 1 and method 2 (due to the empty cluster resulting from method 1, see ***Table S2***, last row, 2nd – 4th column). This further demonstrates the high fluctuation and coincident instability of the inactive system.

Notably, the main clusters' sizes nicely reflect the activity levels by a constant decrease for more fluctuating or inactive systems: main clusters resulting out of method 1 show six to ten members for active or more stable systems (which concerns more than half of all snapshots investigated) and none to five members for inactive systems and the more instable system µ-PIIIA on Kv1.6-5P2 (***Table S2***, 2nd column). Likewise, method 2 yields main clusters of sizes four to eight for active and stable semi-active systems, while consistently giving main clusters of only two members for inactive systems and for µ-PIIIA on Kv1.6-5P2 (***Table S2***, 3rd column). Consequently, the numbers of clusters generated by method 2 increases for inactive and fluctuating semi-active systems and reaches from nine to ten, while decreasing from six to three for stable semi-active and active systems (***Table S2***, 3rd column).
At the same time, however, these numbers also indicate that a more stable system does not necessarily contain a more active substance, as can be seen for example from the mutual comparison of cluster method's 2 results for the three systems µ-PIIIA on Kv1.1, µ-PIIIA Kv1.6-5P1 and µ-SIIIA on Kv1.6 (***Table S2***, 3rd column).

Table S2: main stages of the representative snapshot selection process

	**result cluster analysis 1**	**result cluster analysis 2**	**preselection: intersection cluster (if not empty, union cluster else)**	**"preliminary" representative:** toxin RMSD to avg. toxin structure [Å]	**revision:** RMSD of preliminary representative to toxins of last three sim. snapshots [Å]	**final representative:**
**µ-PIIIA Kv1.6**	{3,4,5,6,7,8,9,10}	{1},{2},{3,4,5, 6,7,8,9,10},{11}	{3,4,5,6,7,8,9,10}	snapshot {5} at 200ns: 1.5248	to snapshot {9} (at 400ns): 2.1996 to snapshot {10} (at 450ns): 2.3839 to snapshot {11} (at 500ns): 4.0344	**snapshot {5} at 200ns**
**µ-PIIIA Kv1.1**	{2,3,4,5,6,7,8,9, 10,11}	{1},{2},{3,5}, {4},{6,10}, {7,8,9,11}	{7,8,9,11}	snapshot {9} at 400ns: 1.8812	to snapshot {9} (at 400ns): 2.0698 to snapshot {10} (at 450ns): 0.0000 to snapshot {11} (at 500ns): 2.5402	**snapshot {9} at 400ns**
**µ-PIIIA Kv1.6-5P1**	{2,5,6,9,10,11}	{1},{2,3}, {4,5,6,7}, {8,9,10},{11}	{5,6}	snapshot {6} at 250ns: 2.5522	to snapshot {9} (at 400ns): 4.8724 to snapshot {10} (at 450ns): 4.3513 to snapshot {11} (at 500ns): 5.0838	**snapshot {6} at 250ns**
**µ-SIIIA Kv1.6**	{2,3,4,5,6,7,8,9, 10,11}	{1},{2,3}, {4,5,6,7,8,9, 10,11}	{4,5,6,7,8,9,10,11}	snapshot {6} at 250ns: 1.2155	to snapshot {9} (at 400ns): 1.6569 to snapshot {10} (at 450ns): 2.4387 to snapshot {11} (at 500ns): 1.9899	**snapshot {6} at 250ns**
**µ-PIIIA Kv1.6-5P2**	{2,3,4,5,6}	{1},{2,6},{3}, {4,5},{7},{8}, {9},{10},{11}	{2,4,5,6}	snapshot {5} at 200ns: 2.2403	to snapshot {9} (at 400ns): 5.0467 to snapshot *{10}* *(at 450ns):* ***7.7670*** to snapshot *{11}* *(at 500ns):* ***8.5439***	**snapshot {10} at 450ns**
**µ-PIIIA Kv1.5**	{4,5,6,7}	{1},{2},{3}, {4,7},{5},{6}, {8},{9},{10}, {11}	{4,7}	snapshot {4} at 150ns: 2.4267	to snapshot {9} (at 400ns): 5.0881 to snapshot {10} (at 450ns): 5.5301 to snapshot {11} (at 500ns): 4.3945	**snapshot {4} at 150ns**
**µ-GIIIA Kv1.6**	{}	{1},{2},{3}, {4},{5},{6},{7}, {8},{9,11},{10}	{9,11}	snapshot {9} at 400ns: 6.5590	to snapshot {9} (at 400ns): 0.0000 to snapshot {10} (at 450ns): 4.2314 to snapshot {11} (at 500ns): 2.7594	**snapshot {9} at 400ns**

3. Analysis of channel-toxin interactions

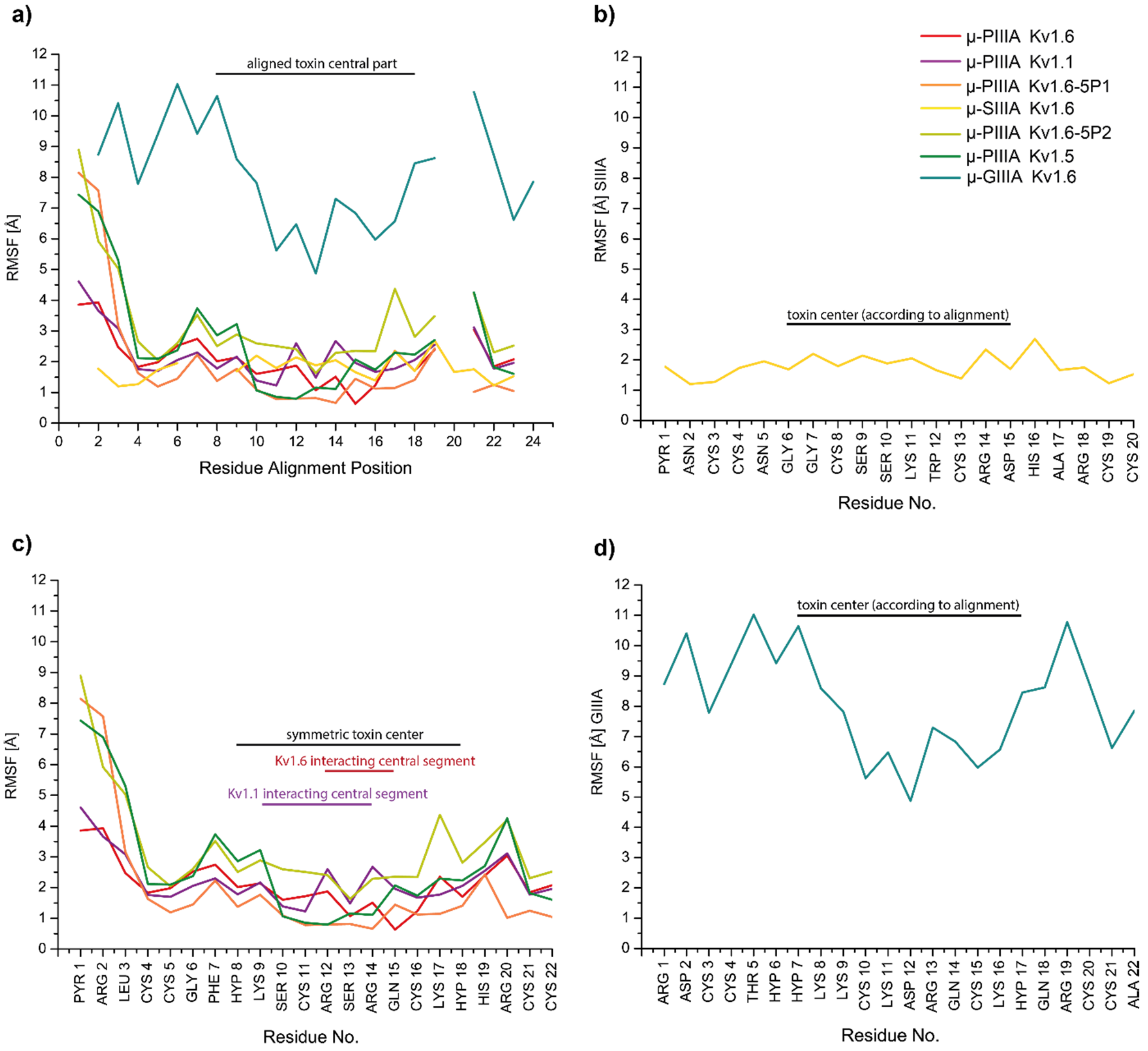

Figure S5: Per residue RMSF summary of the toxins shown per simulation **a)** according to the toxin alignment (Figure 3a); **b)** for simulation μ-SIIIA on Kv1.6 – yellow; **c)** for μ-PIIIA simulations (on Kv1.6 – red, on Kv1.1 – purple, on Kv1.6-5P1 – orange, on Kv1.6-5P2 – yellow-green, on Kv1.5 – dark green), **d)** for μ-GIIIA on Kv1.5 – cyan.

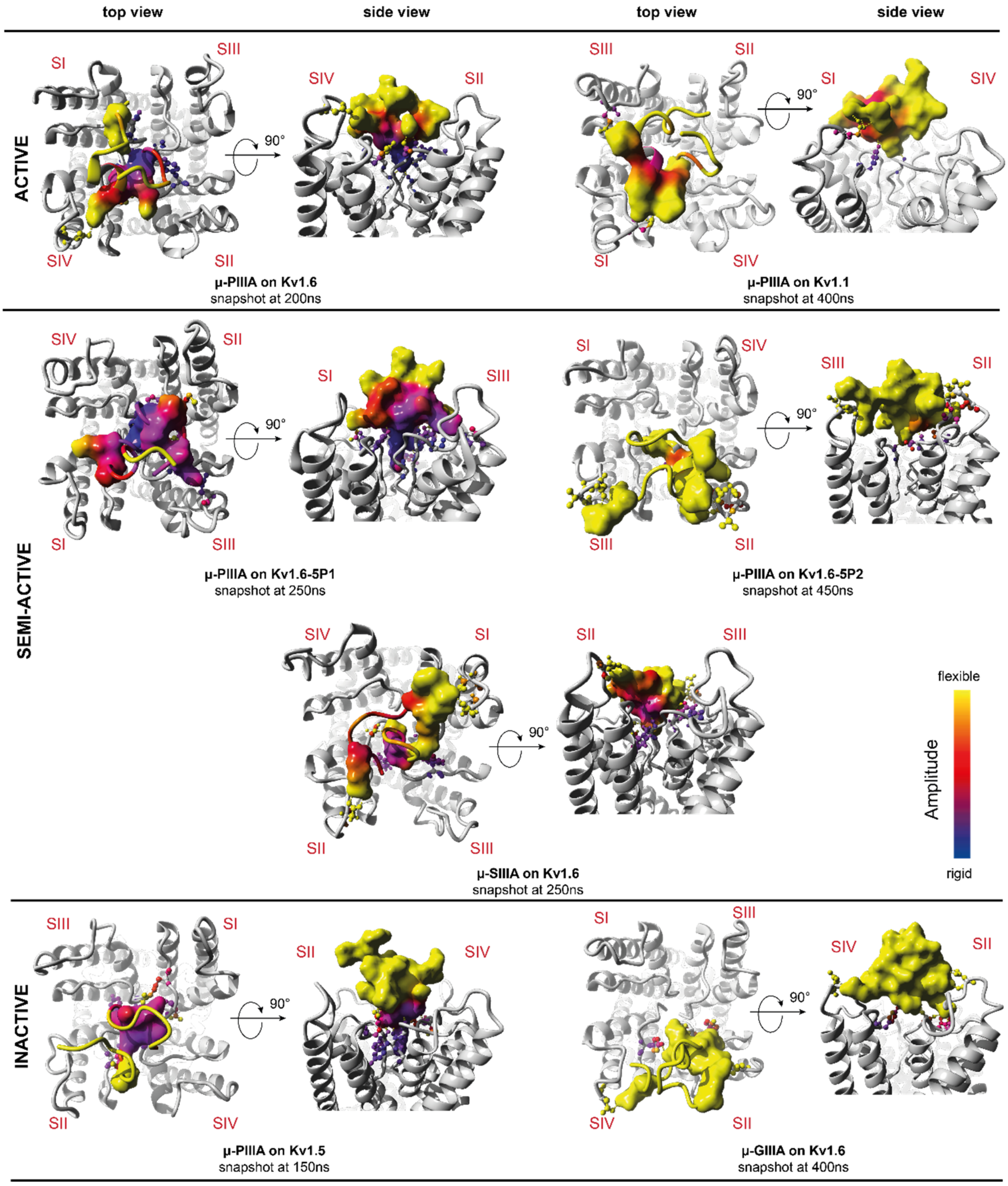

Figure S6: b-Factor coloring of the interacting toxin and channel residues (flexible – yellow, rigid – blue); the toxin is shown partly in molecular surface (interacting residues) and in secondary structure representation (remaining residues) in the top view and in complete molecular surface representation in the side view; interacting channel residues are shown as ball and sticks; remaining channel is shown as secondary structure (gray); the displayed structures are energy-minimized and depict the selected representative snapshots from the simulation data; channel subunits are indicated by Roman numerals (SI – SIV).

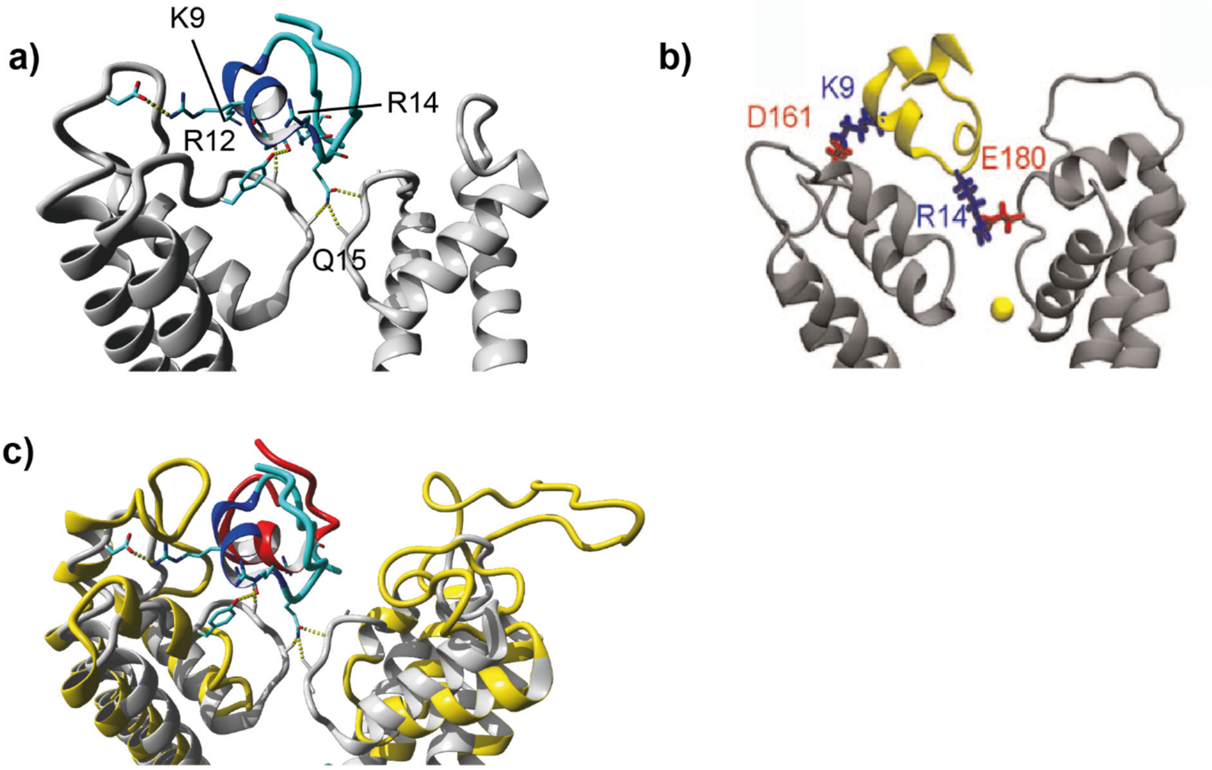

Figure S7: Comparison of predicted μ-PIIIA binding to **a)** K_V1.6 as revealed in this study, **b)** to Na_V1.4 (Fig. adapted from Ref. 29) and **c)** to the experimental cryo-EM structure of the structurally related conotoxin μ-KIIIA (red) bound to Na_V1.2 (yellow, pdb 6J8E, Ref. 11, PIIIA-K_V1.6 is colored as in a)).
